# Can nano-hydroxyapatite permeate the oral mucosa? A histological study using three-dimensional tissue models

**DOI:** 10.1371/journal.pone.0215681

**Published:** 2019-04-23

**Authors:** Shogo Komiyama, Ryosuke Miyasaka, Keiichiro Kikukawa, Roslyn Hayman

**Affiliations:** Sangi Co., Ltd, Central Research Laboratory, Kasukabe, Saitama, Japan; West Virginia University School of Medicine, UNITED STATES

## Abstract

Nano-hydroxyapatite is used in oral care products worldwide. But there is little evidence yet whether nano-hydroxyapatite can enter systemic tissues via the oral epithelium. We investigated histologically the ability of two types of nano-hydroxyapatite, SKM-1 and Mi-HAP, to permeate oral epithelium both with and without a stratum corneum, using two types of three-dimensional reconstituted human oral epithelium, SkinEthic HGE and SkinEthic HOE respectively with and without a stratum corneum. Both types of nano-hydroxyapatite formed aggregates in solution, but both aggregates and primary particles were much larger for SKM-1 than for Mi-HAP. Samples of each tissue model were exposed to SKM-1 and Mi-HAP for 24 h at concentrations ranging from 1,000 to 50,000 ppm. After treatment, paraffin sections from the samples were stained with Dahl or Von Kossa stains. We also used OsteoSense 680EX, a fluorescent imaging agent, to test for the presence of HAP in paraffin tissue sections for the first time. Our results for both types of nano-hydroxyapatite showed that the nanoparticles did not penetrate the stratum corneum in SkinEthic HGE samples and penetrated only the outermost layer of cells in SkinEthic HOE samples without stratum corneum, and no permeation into the deeper layers of the epithelium in either tissue model was observed. In the non-cornified model, OsteoSense 680EX staining confirmed the presence of nano-hydroxyapatite particles in both the cytoplasm and extracellular matrix of outermost cells, but not in the deeper layers. Our results suggest that the stratum corneum may act as a barrier to penetration of nano-hydroxyapatite into the oral epithelium. Moreover, since oral epithelial cell turnover is around 5–7 days, superficial cells of the non-keratinized mucosa in which nanoparticles are taken up are likely to be deciduated within that time frame. Our findings suggest that nano-hydroxyapatite is unlikely to enter systemic tissues via intact oral epithelium.

## Introduction

Nanomaterials are generally defined as entities with at least one dimension in the range of 1–100 nm [1]. In the European Union, nanomaterial has been officially defined as meaning ‘*a natural*, *incidental or manufactured material containing particles*, *in an unbound state or as an aggregate or as an agglomerate and where*, *for 50% or more of the particles in the number size distribution*, *one or more external dimensions is in the size range 1 nm*–*100 nm’* [2].

It is known that the state of electrons on nanomaterials varies with decreasing particle size, giving them different chemical [3, 4], mechanical [5] and optical [6] properties from micromaterials. In recent years, nanotechnology has been applied in various fields using these property changes. Titanium dioxide (TiO_2_) nanoparticles are used to protect the skin against ultraviolet rays in the cosmetics field [7]. In the medical field, the use of biocompatible nanoparticles in drug delivery systems, for example for selective cell-targeting and gene therapy is being widely studied [8].

Nano-hydroxyapatite (n-HAP) is already used widely in the dental field [9]. A naturally occurring mineral represented by the formula Ca_10_(PO_4_)_6_(OH)_2_, it accounts for 97% of tooth enamel and 70% of dentin. Enamel is formed of prisms comprising rod-like n-HAP particles in parallel arrangement. In a healthy oral environment with normal saliva flow, enamel density is relatively stable, with demineralization and remineralization occurring continuously at the tooth surface. Dental caries is an oral disease that occurs when demineralization exceeds the rate of remineralization, causing the collapse of this equilibrium [10]. In recent years, synthetic n-HAP has been increasingly studied as an agent to improve the oral environment. For example, it is reported that artificially created subsurface lesions in extracted human teeth are remineralized by artificial saliva containing n-HAP [11], and that an enamel prism-like structure similar to natural enamel can be constructed by self-assembly of synthesized rod-like n-HAP particles [12]. Toothpaste containing n-HAP which shows clinically a remineralization effect similar to that of fluoride-containing toothpaste is already commercially available [13], and it is reported that dentin hypersensitivity is reduced by occlusion of dentinal tubules by n-HAP particles [14,15].

Widespread discussion on the safety of nanomaterials has arisen, although technologies using nanomaterials are already utilized in our daily life. It is reported that TiO_2_ nanoparticles do not penetrate the stratum corneum of the skin, in both in vivo and in vitro studies [16, 17]. On the other hand, there are some reports that oral administration or intraperitoneal injection of TiO_2_ nanoparticles caused liver damage in mice, and intra-tracheal administration showed TiO_2_ nanoparticles induced dose-dependent inflammatory lesions in rats [18]. It is reported that nano-silica shows different bio-properties with respect to skin penetration and nuclear entry from the micro-level material, and exerts various adverse biological effects at a local and systemic level, such as DNA fragmentation [19].

There is some concern whether n-HAP particles in toothpaste may pass through the oral epithelium and enter into the systemic tissues, because it has been reported that n-HAP particles are taken up and cause cytotoxicity in monolayer cultured human oral epithelial (TR146) cells [20]. However, a series of defense mechanisms operate in the oral cavity against the entry of solid particles, and data obtained in a monolayer cell-culture system are likely to be insufficient to indicate whether n-HAP particles may enter systemic tissue via the oral epithelium. Saliva functions as a first line of defense in the oral cavity against the invasion of harmful substances and microorganisms into the body [21, 22]. It is known that nanoparticles tend to become aggregated by proteins in saliva and to increase in size, and this phenomenon is likely to inhibit cellular uptake [23]. It has been postulated that 50–90% of TiO_2_ nanoparticles aggregated in saliva could not penetrate into the oral epithelium [24]. The mucous gel that forms a composite layer covering the oral epithelium also plays a protective role against entry of solid particles; smaller particles are confined and larger particles are limited in movement by the nanosized pores and/or pockets in the mesh structure formed by the gel [25]. As for the epithelium itself, it is stratified and thick, and its outermost layer subject to constant desquamation, with an estimated turnover of 5–7 days [26]. Unlike the skin, the oral mucosa comprises both cornified and uncornified regions, depending on its location in the mouth [27]. In cornified regions, the stratum corneum or outermost layer is mainly composed of keratin proteins formed by the continuous death of spinous layer cells [28, 29] and this keratinous layer forms part of the oral defense mechanism, as it is known that, for any material to penetrate this layer the ‘500 Dalton rule’ applies, i.e. substances more than 500 Daltons or approximately 1 nm in size cannot penetrate the stratum corneum [30, 31].

As a preliminary step to investigate whether n-HAP particles can enter systemic tissues through the oral epithelium, we studied histologically to what extent n-HAP could penetrate the stratified layers in two types of three-dimensional (3-D) reconstituted human oral epithelial models, one with and one without a stratum corneum, in what we believe to be the first study of its kind using n-HAP particles.

## Materials and methods

### Preparation of n-HAP samples

Two types of n-HAP were used in the present study, both prepared by Sangi Co., Ltd., Japan, one having rod-like nano-scale primary particles and produced by wet chemical synthesis (SKM-1) and the other having smaller, irregularly shapes nanoparticles and produced by similar chemical synthesis followed by grinding in a wet-mill (Mi-HAP).

### Physicochemical evaluation of SKM-1 and Mi-HAP

#### (1) Particle size and morphology

To investigate the primary particle size and morphology of SKM-1 and Mi-HAP, each was observed by transmission electron microscope (TEM: JEM-2100HR, JEOL Ltd., Japan). Samples of each powder were mounted onto a collodion-coated copper grid, after which each sample was observed by TEM at an acceleration voltage of 200 kV and magnification of 200,000 times. The average primary particle size of SKM-1 and Mi-HAP was calculated using 606 particles in 62 fields for the former and 777 particles in 54 fields for the latter, respectively.

SKM-1 and Mi HAP samples were suspended at 50,000 ppm in the maintenance medium (Episkin, France), and the particle size distribution of each sample was measured using a laser diffraction particle size distribution analyzer (LA-950, HORIBA Ltd, Japan).

#### (2) Specific surface area

The specific surface area of each nanomaterial was measured using a surface area and pore size analyzer (SA 3100, Beckman Coulter, Inc, USA) using N_2_ as adsorbent at −196°C after outgassing the samples for 20 min at 120°C. The Brunauer-Emmett-Teller (BET) specific surface area was calculated for each from the N_2_ adsorption isotherm. The amount of each sample used for specific surface area measurement was approximately 0.1 g, and both samples were measured in triplicate.

#### (3) Zeta potential

Measurement of zeta potential was outsourced to Shimadzu Techno-Research, Inc. SKM-1 and Mi-HAP were each suspended at 1,000 ppm in the maintenance medium and the pH of each suspension immediately recorded. Samples were collected in a capillary cell and the zeta potential was measured using a zeta potential analyzer (Zetasizer ZS, Malvern Instruments, UK). The zeta potential and pH were measured in duplicate.

### Mucosal permeability testing

Two types of 3-D oral mucosal tissue models were used to investigate the permeability of each n-HAP sample into the oral epithelium, one a human gingival epithelial model with stratum corneum (SkinEthic HGE) and the other a human oral epithelial model without stratum corneum (SkinEthic HOE) (Episkin, France). These tissue models were cultured with SkinEthic maintenance medium according to the manufacturer’s directions. After preincubation, each insert dish with the 3-D tissue was moved to a 24-well multi-plate previously filled with 300 μL of maintenance medium. SKM-1 and Mi-HAP were respectively suspended in maintenance medium at different concentrations, and 50 μL of each respective suspension added to an insert dish. Plates were then incubated at 37°C in a 5% CO_2_ atmosphere for 24 h. After incubating, all 3-D cultured tissues were fixed in Lilly's buffered formalin solution at 4°C for 24 h and removed from the insert dish carefully together with the polycarbonate filter in each case, using a micro-knife. The tissues were dehydrated in an alcohol series and xylene and then embedded in paraffin according to the conventional method. Slides were prepared by slicing at a thickness of 3 μm.

The doses of SKM-1 and Mi-HAP were calculated as follows. Collins et al reported that the total surface area of the adult oral cavity is 214.7 ± 12.9 cm^2^ [32]. Presuming that adults use 1 g of toothpaste containing 10% n-HAP in one brushing, the net amount of exposure to n-HAP would be 100 mg, or on a per unit area basis 465.7 ± 26.3 μg/cm^2^ using those two values. This calculated value corresponds to about 5,000 ppm of n-HAP when suspended in 50 μL of maintenance medium fluid. Based on this concentration, the doses of n-HAP were set at 0, 1,000, 5,000, 10,000 and 50,000 ppm in 50 μL of medium, with medium only (zero n-HAP) added to insert dishes as a negative control. Each test group consisted of 3 tissue samples.

### Detection of n-HAP particles by histochemical and fluorescent methods

All tissue sections, including negative controls, were stained by Dahl [33] and Von Kossa [34] methods for histochemical detection of SKM-1 and Mi-HAP. Calcium deposits in tissue stain reddish orange with alizarin red S, contained in Dahl staining solution, and calcium phosphate and calcium carbonate deposits stain black or blackish brown with Von Kossa stain. After staining, each tissue section was observed using an upright microscope (BX 53, Olympus Corporation, Japan) at a magnification of 600 times.

As a new and potentially clearer method of investigating the localization of n-HAP in histological samples, a further group of paraffin sections of SkinEthic HOE tissue were stained with OsteoSense 680EX (PerkinElmer, Inc., USA), a fluorescent dye commonly used in in vivo applications because of its affinity for HAP. OsteoSense 680EX is usually administered intravascularly for the imaging of bone resorption and/or regeneration sites because it binds specifically to biological HAP [35, 36]. However, there has been no previous report using OsteoSense 680EX histologically, and we believe our study is the first to test its ability to detect synthesized n-HAP in paraffin tissue sections.

First, we tested whether OsteoSense 680EX could bind to the chemically synthesized n-HAP used in our study. SKM-1 and Mi-HAP were respectively mixed with a solution of OsteoSense 680EX prepared to be 0.08 nmol/mL in DW, and each mixture was stirred at 37°C for 3 h. Each sample was then centrifuged at 12,000 rpm for 10 min and the precipitate washed in DW and treated for 5 min with an ultrasonic device (USC-100Z38S-22, Ultrasonic Engineering Co., Ltd., Japan), and this procedure was repeated 3 times. The precipitate of SKM-1 or Mi-HAP was then suspended in a small amount of DW and a drop of each suspension placed on a slide glass and sealed with glycerol (Wako, Japan). As a negative control, DW alone was used without addition of OsteoSense 680EX under the same conditions.

In a preliminary experiment using n-HAP exposed tissue, no red fluorescence after staining with OsteoSense 680EX could be detected. We suspected that the cause may be masking of n-HAP by the presence of protein and found that after pre-treatment of the tissue with trypsin, a routine procedure used in immunostaining, HAP could be detected in the tissue by staining with OsteoSense 680EX.

As a result, each paraffin section, after deparaffinization, was first immersed in a trypsin solution (TRYPSIN 1: 250, Difco Laboratories, USA) then prepared to be 0.01% in DW at 37°C for 3 min, and washed 3 times with DW. To stop further digestion by trypsin, each section was then immersed in a trypsin inhibitor solution (Trypsin Inhibitor from Soybean, Wako, Japan) prepared to be 0.1% in DW at 37°C for 15 min, then washed 3 times with DW. Tissue sections were then incubated in 150 μL of 0.08 nmol/mL OsteoSense 680EX at 37°C for 3 h. After incubating, each section was washed with running water for 5 min, and then with DW 3 times. Water was removed as much as possible from the slide and each tissue sealed with glycerol. All sections were observed using a confocal laser scanning microscope (Leica Microsystems, TCS-SP 5, Germany) under the following conditions: excitation wavelength 633 nm; emission wavelength 680 ± 10 nm; magnification of 630 times.

## Results

### TEM observation of n-HAP samples

**Fig 1:**
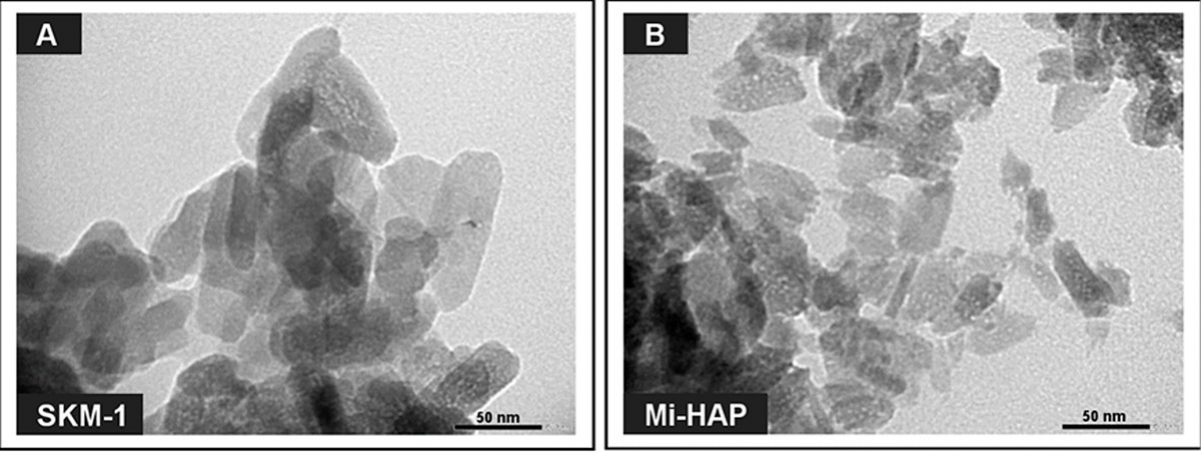
TEM images of SKM-1 and Mi-HAP. SKM-1 and Mi-HAP are shown in A and B, respectively. SKM-1 was rod-like in shape, with an average primary particle size of 72 nm × 15 nm. Mi-HAP was irregularly shaped, with an average primary particle size of 53 nm × 9 nm.

TEM images of SKM-1 and Mi-HAP (magnification ×200,000) are shown in Fig 1. SKM-1 was observed to be rod-like in shape, with an average primary particle size of 72 nm × 15 nm (Fig 1A). Mi-HAP was seen to be irregular in shape, with an average primary particle size of 53 nm × 9 nm (Fig 1B).

### Specific surface area, particle size and zeta potential

The average values of specific surface area, particle size, zeta potential of SKM-1 and Mi-HAP and the pH values of their suspension are shown in Table 1. The specific surface area of Mi-HAP was about three times larger than that of SKM-1. The results of particle size distribution analysis in the maintenance medium showed that SKM-1 formed aggregates at micron level and Mi-HAP formed aggregates at nano-level. Mi-HAP had a larger negative charge than SKM-1 and the pH value of the Mi-HAP suspension was higher than that of the SKM-1 suspention.

**Table 1:** Chemical properties of the two types of n-HAP in culture medium.

	SKM-1	Mi-HAP
Specific surface area (m^2^/g)	47.9	140.3
Particle size (nm)	7489	95
Zeta potential (mV)	−14.6	−41.3
pH	8.0	9.2

### Histological evaluation

**Fig 2:**
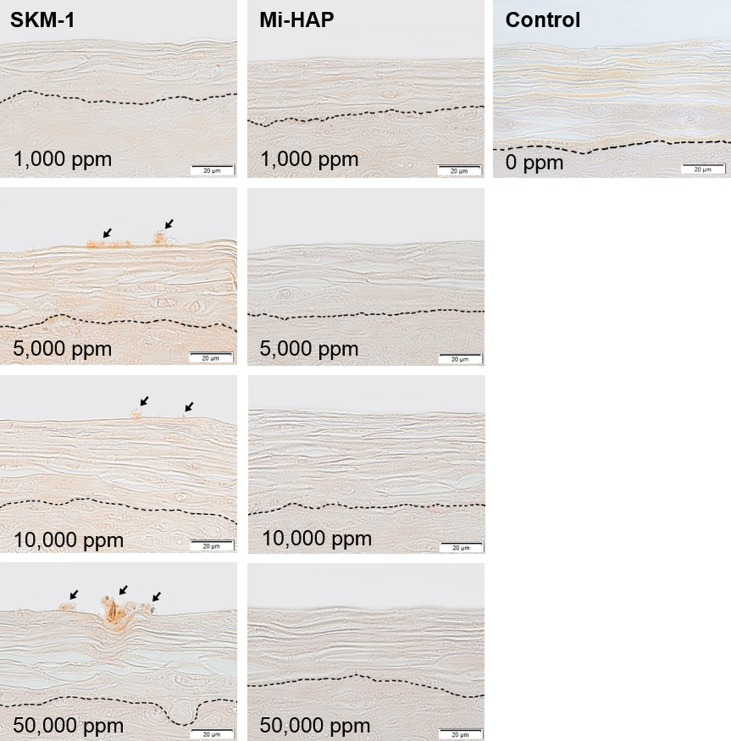
SkinEthic HGE tissue after exposure to n-HAP (Dahl stain). Left and middle columns show tissues exposed to SKM-1 and Mi-HAP, respectively. Right column shows control tissue. The broken line shows the boundary between the stratum corneum and non-keratinized stratified squamous epithelium. Numbers show the concentration of n-HAP applied, and bar shows scale (20 μm). Calcium-containing deposits (stained reddish orange; arrows) were observed on the surface of the stratum corneum in the SKM-1 group, at concentrations of 5,000 ppm or more, but not within the stratum corneum or the underlying stratified cellular layer. In contrast, tissues exposed to Mi-HAP were negative for the stain at all concentrations.

**Fig 3:**
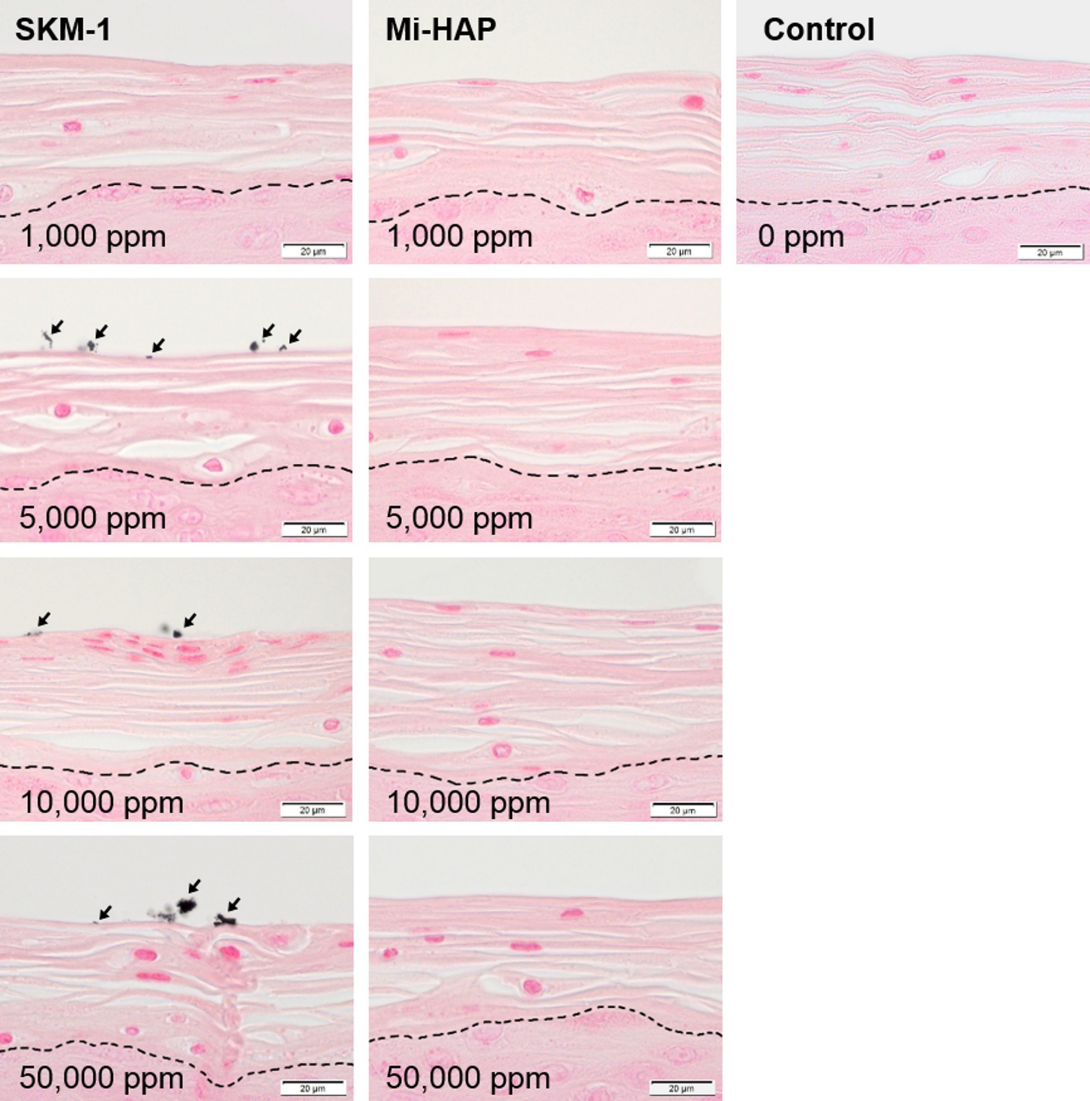
SkinEthic HGE tissue after exposure to n-HAP (Von Kossa stain). Left and middle columns show tissues exposed to SKM-1 and Mi-HAP, respectively. Right column shows control tissue. The broken line shows the boundary between the stratum corneum and non-keratinized stratified squamous epithelium. Numbers show the concentration of n-HAP applied, and the bar shows scale (20 μm). Calcium-containing deposits (black stain; arrows) were observed on the surface of the stratum corneum in the SKM-1 group, at concentrations of 5,000 ppm or more, but not within the stratum corneum or the underlying stratified cellular layer. In contrast, tissues exposed to Mi-HAP were negative for the stain at all concentrations.

Images of SkinEthic HGE tissue stained by Dahl and Von Kossa methods are shown in Figs 2 and 3 respectively. All sections in the control groups without addition of n-HAP showed a negative reaction to the stains.

In the SkinEthic HGE group stained with Dahl (Fig 2), several aggregates stained reddish orange were observed on the surface of the stratum corneum in the SKM-1 group at concentrations of 5,000 ppm or more (arrows), but not within the stratum corneum or the squamous epithelium. In the case of epithelium exposed to Mi-HAP, positive staining was not observed on or within the tissue at any concentration.

Von Kossa stained images of SkinEthic HGE tissue are shown in Fig 3. The presence of calcium-containing deposits (stained black; arrows) was observed on the surface of the stratum corneum in the SKM-1 group at concentrations of 5,000 ppm and more, but not within the stratum corneum or the squamous epithelium. In contrast, no black staining was observed at any concentration in the Mi-HAP group. The histological findings observed in the SkinEthic HGE sections stained with Von Kossa were similar to those observed with Dahl stain.

**Fig 4:**
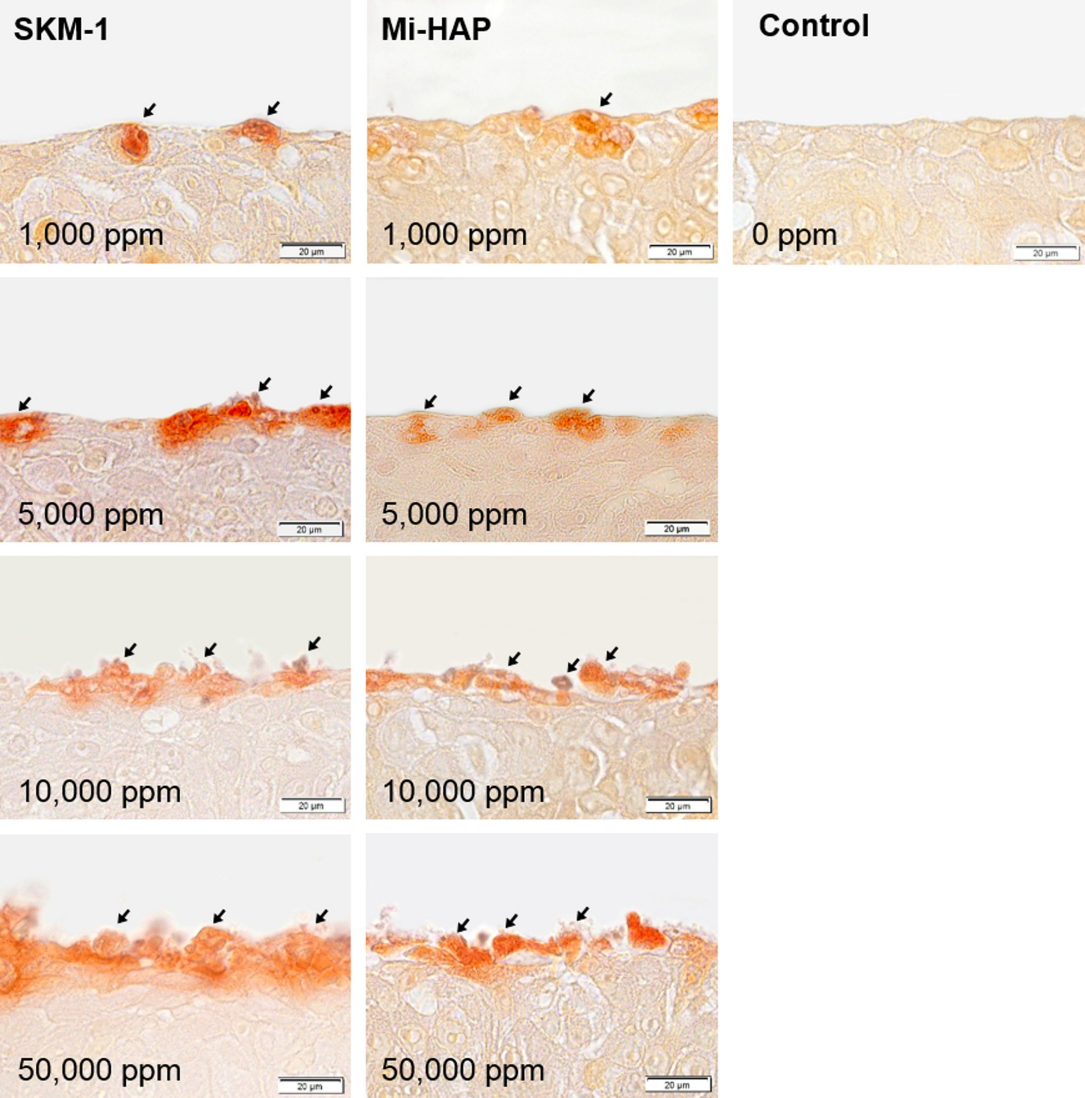
SkinEthic HOE tissue after exposure to n-HAP (Dahl stain). Left and middle columns show tissues exposed to SKM-1 and Mi-HAP, respectively. Right column shows control tissue. Numbers show the concentration of n-HAP applied, and the bar shows scale (20 μm). Calcium-containing deposits (stained reddish orange; arrows) were observed in the cytoplasm of outermost epithelial cells in both groups from 1,000 ppm, and the extent of positive staining was concentration-dependent. However no positive reaction in the deeper layers of tissue in either group was observed.

In contrast, in the SkinEthic HOE group, without stratum corneum (Fig 4), positive reddish orange staining by Dahl was observed in the cytoplasm of superficial epithelial cells at concentrations of 1,000 ppm and above in tissue exposed to both SKM-1 and Mi-HAP. The extent of positive reaction increased with increasing concentrations of n-HAP. Positive staining was stronger in tissues exposed to SKM-1 than in tissues exposed to Mi-HAP, and a diffuse reaction around superficial cells was observed in the SKM-1 group at a concentration of 50,000 ppm. However, positive staining was not detected in the deeper layer of stratified cells in either group.

**Fig 5:**
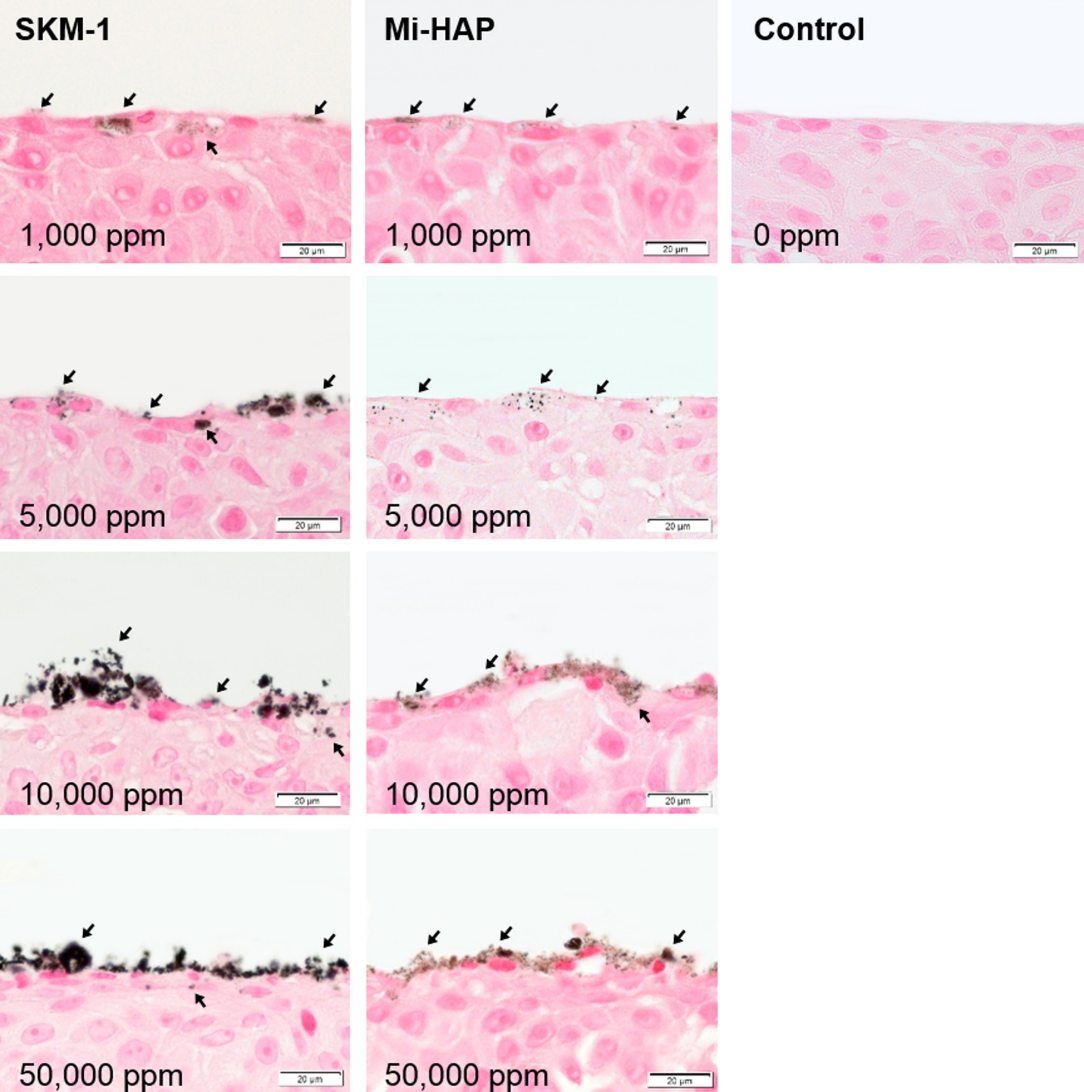
SkinEthic HOE tissue after exposure to n-HAP (Von Kossa stain). Left and middle columns show tissues exposed to SKM-1 and Mi-HAP, respectively. Right column shows control tissue. Numbers show the concentration of n-HAP applied, and the bar shows scale (20 μm). Black or blackish brown-stained deposits (arrows) were seen on the surface and within the cytoplasm of cells in the outermost epithelial layer at all concentrations in both the SKM-1 and Mi-HAP groups, and the extent of positive staining was concentration-dependent, but was less in the Mi-HAP group than in the SKM-1 group. However positive staining was not observed in the deeper layers of tissue in either group.

Von Kossa stained images of SkinEthic HOE tissue, without stratum corneum, are shown in Fig 5. Calcium-containing black or blackish brown deposits were observed on the surface and within the cytoplasm of cells of the outermost epithelial layer both in the SKM-1 and Mi-HAP groups, at concentrations of 1,000 ppm or more, and the amount of deposits was concentration-dependent, though the degree of positive staining was less in the Mi-HAP group than in the SKM-1 group. However, black or blackish brown deposits were not detected in the deeper layer of stratified cells in any group, similar to the result observed with Dahl staining.

### Evaluation of fluorescent staining

**Fig 6:**
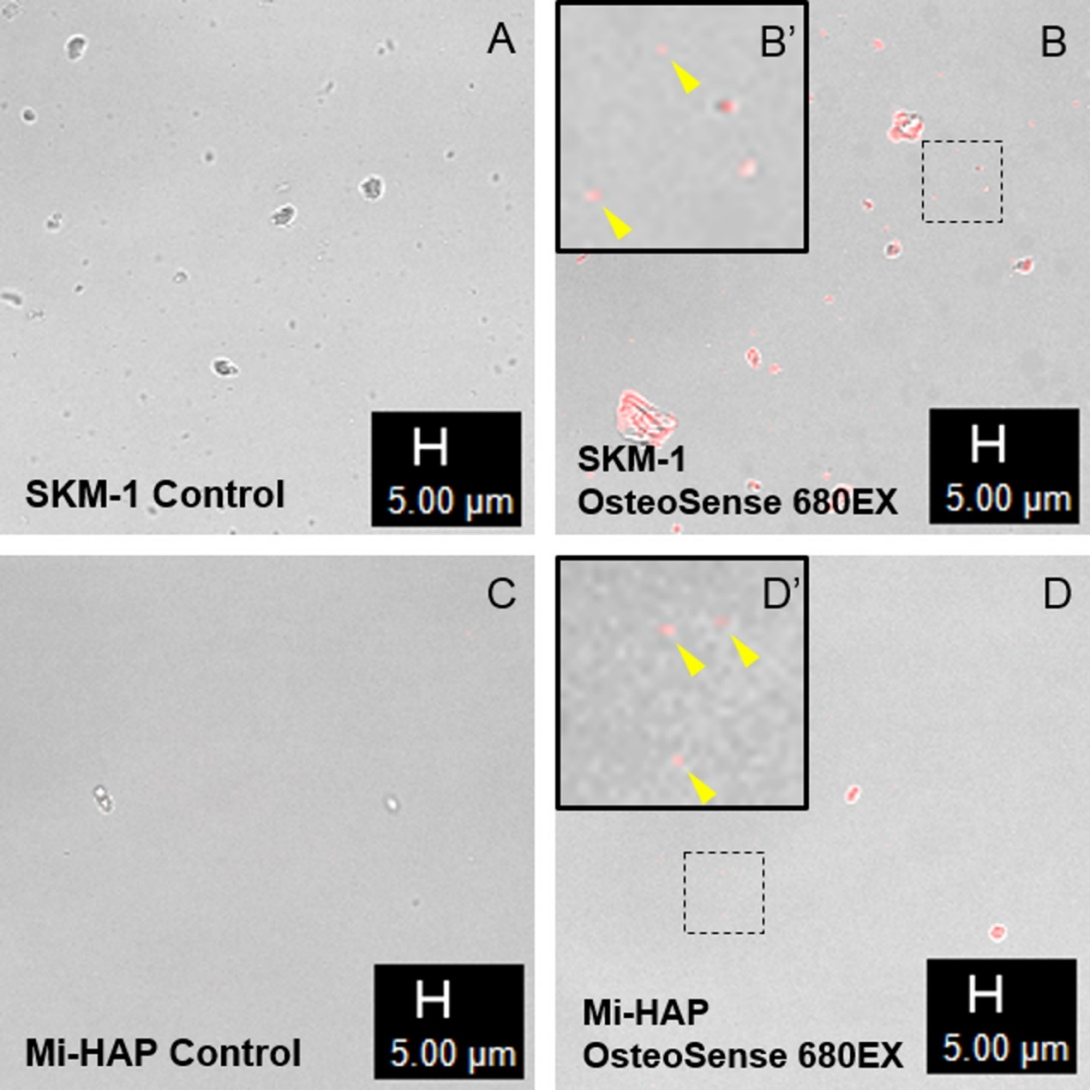
n-HAP powders before and after staining with OsteoSense 680EX. Confocal laser scanning micrographs of SKM-1 (A and B) and Mi-HAP (C and D) before and after treatment respectively with OsteoSense 680EX fluorescent stain; B’ and D’ show higher magnification of the areas surrounded by a dotted line. The black bar shows scale (5 μm). Powders untreated with OsteoSense 680EX (A, C) showed no autofluorescence at an excitation wavelength of 680 nm, but after treatment with OsteoSense 680EX (B, D), powders exhibited red fluorescence at the same wavelength, indicating that OsteoSense 680EX bound well to the n-HAP particles (B’, D’: arrowheads).

**Fig 7:**
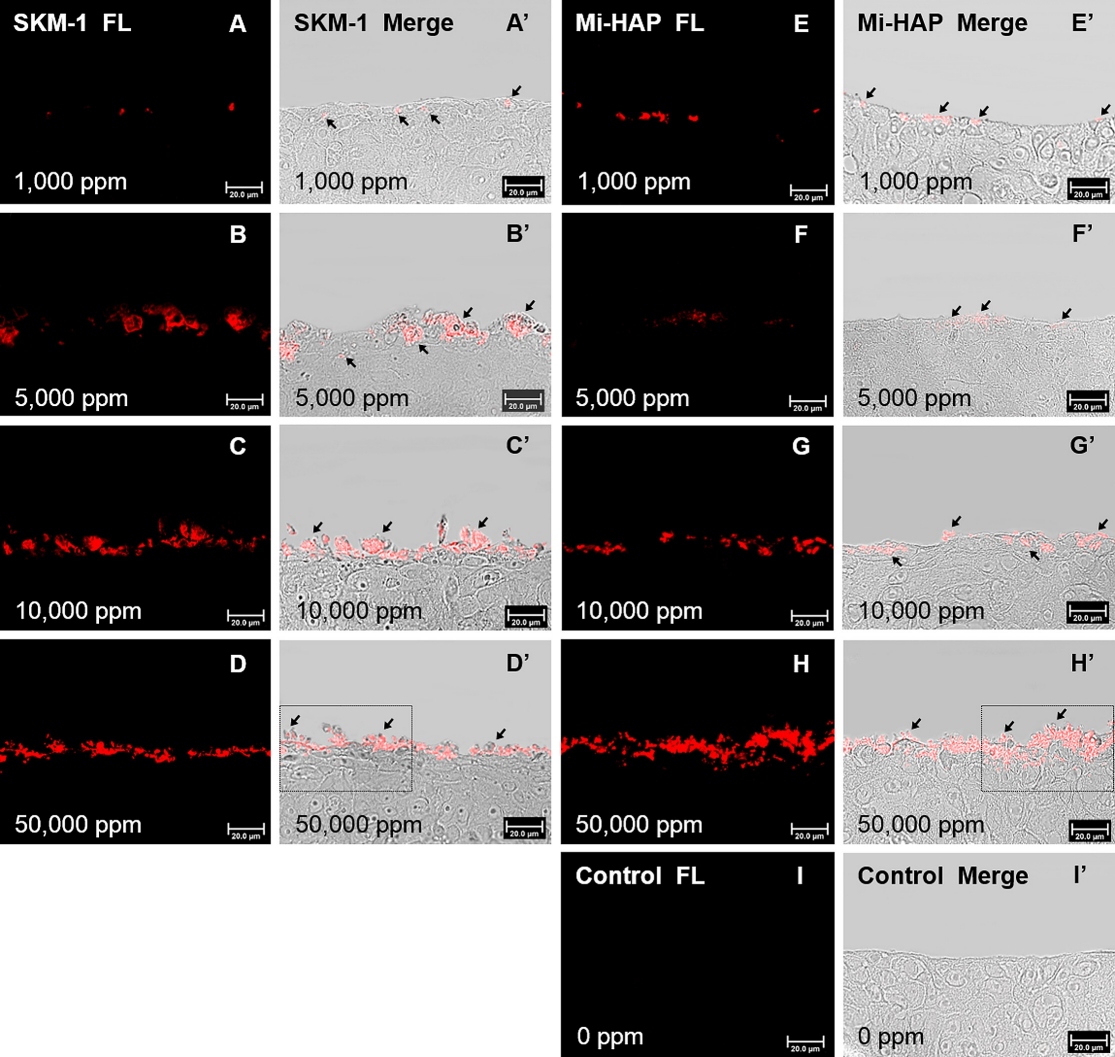
SkinEthic HOE tissue treated with OsteoSense 680EX after exposure to n-HAP. A–D and E–H show dark field images, and A’–D’ and E’–H’ show merged images of the dark field and the bright field for tissues exposed respectively to SKM-1 or Mi-HAP. I and I’ show control tissue. Numbers show the concentration of n-HAP applied and the bar shows scale (20 μm). The location of n-HAP in the epithelium indicated by red fluorescence in dark field images can be confirmed by comparison with the respective merged images. Both types of n-HAP were found on the surface and/or in the cytoplasm of cells in the outermost layer at concentrations of 1,000 ppm or more. Mi-HAP showed stronger fluorescence than SKM-1 in the outermost cells and immediately underlying cells, at the highest concentration (50,000 ppm), but fluorescence was stronger for SKM-1 at 5,000 and 10,000 ppm. However for both types of n-HAP no fluorescence in the deeper layers of the tissue at any concentration was observed. Enlargements of the areas surrounded by dotted lines in images D’ and H’ are shown Fig 8 (magnification ×1,260).

Histological evaluation using Dahl and Von Kossa stains, which detect the presence of calcium, suggested that n-HAP particles could not penetrate the stratum corneum or enter the underlying non-keratinized stratified squamous epithelium of the tissue models tested. We attempted to confirm this, and investigate the localization of n-HAP particles in the non-keratinized oral epithelium more clearly, by using OsteoSense 680EX, a fluorescent dye known to bind specifically to HAP in biological tissues, and the results are shown in Figs 6 and 7.

First, preliminary testing confirmed that OsteoSense 680EX could bind to chemically synthesized n-HAP (Fig 6). Confocal laser scanning microscopy showed autofluorescence in the case of both SKM-1 and Mi-HAP respectively (B, D) at the excitation wavelength of 680nm, on treatment with OsteoSense 680EX, compared with untreated controls (A, C).

**Fig 8:**
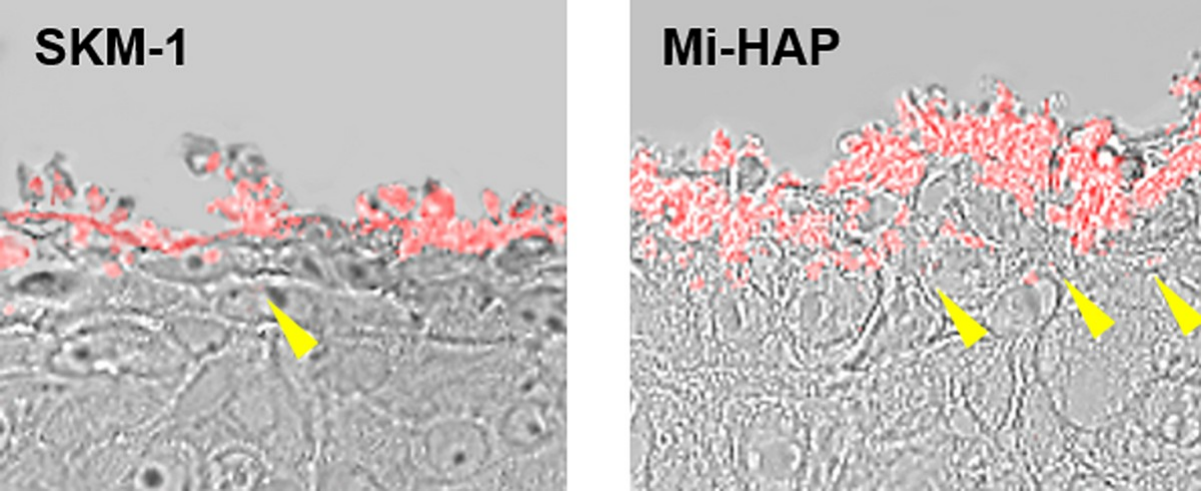
Enlargement of the areas surrounded by dotted lines in Fig 7D’ and 7H’. Higher magnification confirmed that OsteoSense 680EX-positive n-HAP was located on the surface and in the cytoplasm of cells only in the outermost layers, occasionally reaching the second-most layer, in the case of SKM-1 (left: arrowhead), and the third layer and surrounding extracellular matrix in the case of Mi-HAP (right: yellow arrowheads). But no positive reaction in the underlying layers for either type of n-HAP was seen.

Images of SkinEthic HOE tissue treated with n-HAP then stained with OsteoSense 680EX are shown in Fig 7. Fluorescence was detected in the cytoplasm of cells in the outermost layer in all tissues at concentrations of 1,000 ppm or more, and the intensity of fluorescence was concentration-dependent, as seen previously with Dahl and Von Kossa staining. However no fluorescence was detected in the deeper layers of the epithelium in either group. Fig 8 shows at higher magnification (magnification ×1,260) the area surrounded by dotted lines in Fig 7D’ and 7H’. Quantities of both n-HAP aggregates were found in the outermost layer of epithelial cells, and a small amount of fluorescence was also observed in the cytoplasm, and in the case of Mi-HAP, also in the extracellular matrix of the second layer of cells, with a much larger amount of deposits observed in the case of Mi-HAP than for SKM-1. However, no fluorescence was found in the deeper layers of cells for either type of n-HAP when observed at higher magnification.

## Discussion

Recently nanomaterials have come to be used in various fields and the use of nanotechnology in oral care products is increasing worldwide. However, there is still insufficient data on the behavior of nanomaterials in the oral cavity, including whether nanoparticles could enter the bloodstream and systemic tissues via the oral mucosa. We focused on toothpaste which accounts for a large share of oral care products. Toothpastes containing HAP, which is the main component of tooth enamel, are now widely available some of them containing n-HAP. We carried out what we believe to be the first study of its kind using 3-D reconstituted oral mucosal tissue to examine histologically whether n-HAP particles can permeate the oral mucosa, and our results suggest that n-HAP is unlikely to enter the systemic tissues via this route.

According to one generally accepted EU definition, even aggregates are classified as nanomaterials if the primary particle size is in the range of 1–100 nm [37].Two types of n-HAP, SKM-1 and Mi-HAP, were used in this study. SKM-1 showed a larger primary particle size and a smaller negative surface charge than for Mi-HAP. It is known that specific surface area increases as primary particle size decreases [38]. The average value of specific surface area of Mi-HAP was much larger than that of SKM-1, which supported the results of the primary particle size evaluation by TEM.

SkinEthic HGE with stratum corneum and SkinEthic HOE without stratum corneum were used as 3-D oral mucosal tissue models in this study. The exposure amounts of SKM-1 and Mi-HAP nanoparticles administered were calculated based on the estimated surface area of the oral cavity and the amount of toothpaste normally used, to emulate actual likely exposure during tooth brushing, similar to the calculation used by Scheel and Hermann in an earlier study that tested the penetrability of n-HAP in a 3-D human corneal epithelial tissue model (SkinEthic HCE) [39]. Our study however is the first to test n-HAP penetrability using reconstituted 3-D human oral mucosal tissue.

Histological staining by Dahl and Von Kossa in the present study identified calcium deposits in the 3-D tissue exposed to both kinds of n-HAP, but not in untreated control tissue. It is known that HAP dissolves below around pH 5, the most frequently reported value being pH 5.5 [40]. Both types of n-HAP in our study can therefore be presumed to have remained in the form of solid particles when applied to the tissues, since the pH value of the SKM-1 and Mi-HAP suspensions in maintenance medium in both cases was 7.5.This supports the view that the positive reaction observed with Dahl and Von Kossa staining reflected the presence of n-HAP particles. Moreover, the positive reactions observed with both stains were at the same locations in the treated tissues.

In the case of SkinEthic HGE tissues, attachment of Dahl- and Von Kossa-positive deposits on the outermost layer of the stratum corneum was observed only for SKM-1 at 5,000 ppm or more, and no positive reaction within the stratum corneum or in the spinous layer was observed for either type of n-HAP. The reason for this adhesion of SKM-1 but not Mi-HAP to the surface layer of the stratum corneum is unclear. Any n-HAP particles merely precipitating on the surface of the stratum corneum would be likely to be washed away during preparation of the paraffin blocks, so the fact that deposits were observed in the final tissue section indicates strong adhesion to the superficial layers of the stratum corneum. The stratum corneum of the oral mucosa is comprised mainly of keratin proteins rich in glycine, serine, leucine and glutamic acid, formed by the continuous emergence and death of underlying spinous layer cells [26]. Bulk hydroxyapatite is known to adsorb many kinds of proteins depending on their type and physical properties, and environmental factors etc [41]. However little is known about the protein adsorption properties of n-HAP. SKM-1 and Mi-HAP differed not only in size but also in zeta potential, and we postulate that the larger absolute value of surface charge shown in Mi-HAP may be one reason why attachment of Mi-HAP on the surface of the stratum corneum was not observed. The fact that neither type of n-HAP was observed within the stratum corneum or in the underlying spinous layer is supported by the ‘500 Dalton rule’, according to which substances of more than 500 Daltons, or approximately 1 nm in diameter, cannot permeate into the stratum corneum of human skin [30, 31].

In the non-keratogenic SkinEthic HOE model, although positive reactions to Dahl and Von Kossa staining were observed in the cytoplasm of the superficial layer of cells at all concentrations of both SKM-1 and Mi-HAP, the positively stained area was diffuse in the case of Dahl, extending to the cytoplasm and extracellular matrix with increasing concentrations of n-HAP, and the outline of deposits was unclear, whereas in the case of Von Kossa, the positive reaction was observed merely as large black or blackish brown aggregates. Moreover since both stains are used to detect the presence of calcium, the fact that the deposits comprised n-HAP could only be postulated but not confirmed.

However using the fluorescent dye OsteoSense 680EX, which has hitherto been used to detect HAP specifically in in vivo applications, we observed a positive fluorescence reaction in the SkinEthic HOE tissue sections at exactly the same locations and intensity as seen with Dahl and Von Kossa stains, confirming the presence of n-HAP and showing for the first time that OsteoSense 680EX can be used to detect synthetic HAP in histological specimens.

Although the 3-D reconstituted human oral mucosa used in our study closely resembled the cellular structure of the oral mucosa in vivo, environmental factors operating in the oral cavity were obviously not present. Salivary secretions contain substances with many different functions, including minerals, proteins such as immunoglobulins, enzymes, mucins and nitrogen compounds [42]. Little is known about the defense properties of saliva against solid particles, however the attachment of proteins from body fluids such as saliva (the so-called ‘protein corona’) is known to cause aggregation of nanoparticles, increasing their size and altering their surface functions, resulting in a reduction in the amount of nanoparticles taken up into cells [23]. As with other body portals (the airways, female reproductive tract etc.) a complex layer of mucus on the surface of the oral epithelium [43, 44] approximately 70–100 μm thick [45] and containing highly glycosylated mucin fibers, acts as a barrier to trap nanoparticles with viscoelasticity. Because this mucus is supplied from saliva, its turnover time is short and the trapped particles are reported to be removed within minutes [43, 46], suggesting that at least a portion of any n-HAP present in the oral cavity may be trapped by mucus and not permeate into the epithelial cell layers.

In areas of the oral epithelium without stratum corneum, even if nanoparticles penetrate the mucus defense mechanism and reach epithelial cells, further barriers lie in wait. It is known that non-keratinized oral epithelium is thicker than epithelium with stratum corneum, comprising tissue approximately 500–800 μm in thickness [47]. Cells progressively differentiating upwards from the basal layer to the superficial layer in this epithelium change in morphology with differentiation, and it is known that lipids derived from membrane coating granules in the upper third of the mucosa form a barrier against permeation of foreign entities [48]. Furthermore, cells are constantly renewed, and the turnover of epithelial cells from the basal to the superficial layer is reported to be 5–7 days [48], indicating that any superficial oral epithelial cells that are penetrated by nanoparticles would be sloughed within this time frame. We found that there was no n-HAP penetration into the deeper layer of epithelial cells even at an exposure level 10 times higher than what could be considered a normal level during regular toothbrushing.

In addition, while the exposure dosages we used were based on actual likely exposure to nanoparticles from a toothpaste per unit area of the oral cavity, the exposure time was 24 h in this study, whereas in actual toothbrushing, the recommended time is roughly 2–3 min, and the majority of toothpaste is washed out from oral cavity after brushing [49]. Therefore, the level of exposure to n-HAP in this study was much larger than would be the case during actual use of an n-HAP-containing toothpaste. The very small amount of exposure during actual toothbrushing would therefore further reduce the possibility of n-HAP passing through the mucosa to enter the systemic tissues. For these reasons, it was presumed that n-HAP would not enter the systemic tissues via intact oral epithelium. However, it is reported that silver nanoparticles penetrate wounded skin more easily than intact skin [50]. This raises the possibility that n-HAP particles may enter systemic tissue via the wounded oral mucosa. On the other hand, it is reported that n-HAP particles administered intravenously at 300 mg/kg or less in rats did not produce side effects [51], and HAP-sol injected intravenously at 26 mg/kg in rats and dogs showed no chronic damage or permanent side effects over two years of experiments [52]. This suggests that even if n-HAP particles did enter systemic tissue via the wounded oral mucosa, they may not show side effects. However further study is required to determine whether n-HAP particles could enter into systemic tissue via the wounded oral mucosa.

## Conclusion

This study was a first-step experiment to investigate whether n-HAP used in oral care products is likely to enter the systemic tissues via the oral mucosa. Histological investigation showed that neither of the two different types of n-HAP particles used in our study penetrated the stratum corneum of the 3-D oral epithelial model with stratum corneum that we used, though nanoparticles of both types were observed in the cytoplasm and around the membrane of cells in the outermost layers of the 3-D oral epithelial model without stratum corneum, regardless of their size or concentration. Moreover in no case was the presence of n-HAP detected in the deeper layers of the epithelium in either model. In the actual oral mucosa, there are defense mechanisms at work, such as salivary mucin, the mucus membrane and certain barrier functions of mucosal epithelial cells, which are not present in the 3-D reconstituted tissue models. Furthermore, since the exposure dosage of n-HAP used in this study was much larger than the likely exposure during actual toothbrushing, it was concluded that n-HAP particles are very unlikely to enter the blood stream or systemic tissue via intact oral mucosa.

## References

[1] Sergey VD. Nanodimensional and nanocrystalline apatites and other calcium orthophosphates in biomedical engineering. Biology and Medicine. Materials. 2009; 2: 1975–2045. 10.3390/ma9090752 PMC5457100 28773871

[2] Janez P. Commission recommendation of 18 October 2011 on the definition of nanomaterial. OJ L. 2011; 275: 38–40. Available from: https://ec.europa.eu/research/industrial_technologies/pdf/policy/commission-recommendation-on-the-definition-of-nanomater-18102011_en.pdf.

[3] Cristina B, Ivan IP, Kevin R. Nanomaterials and nanoparticles: Sources and toxicity. Biointerphases. 2007; 4: MR17–MR172. 10.1116/1.2815690 20419892

[4] Santosh BS, Praveen KT. Catalysis: A brief review on nano-catalyst. Energy Sci Eng. 2014; 3: 106–115.

[5] David R, Steve M, Shong Y, Rouin F and Vivek S. An ink-jet-deposited passive component process for RFID. IEEE Trans Electron Devices. 2004; 51: 1978–1983.

[6] Seung HK, Heng P, Costas PG, Christine KL, Jean MJ F and Dimos P. All-inkjet-printed flexible electronics fabrication on a polymer substrate by low-temperature high-resolution selective laser sintering of metal nanoparticles. Nanotechnology. 2007; 18: 345202.

[7] Silpa R, Shoma J, Sumod US, Sabitha M, Nanotechnology in cosmetics: Opportunities and challenges. J Pharm Bioallied Sci. 2012; 4: 186–193. 10.4103/0975-7406.99016 22923959 PMC3425166

[8] You HB, Kinam P. Targeted drug delivery to tumors: Myths, reality and possibility. J Control Release. 2011; 153: 198–205. 10.1016/j.jconrel.2011.06.001 21663778 PMC3272876

[9] Pepla E, Besharat LK, Palaia G, Tenore G, Migliau G. Nano-hydroxyapatite and its applications in preventive, restorative and regenerative dentistry: a review of literature. Annali di Stomatologia 2014; 5: 108–114. 25506416 PMC4252862

[10] Featherstone JDB. Dental caries: A dynamic disease process, Australian Dental Journal 2008; 53: 286–291 10.1111/j.1834-7819.2008.00064.x 18782377

[11] Swarup JS, Rao A. Enamel surface remineralization: Using synthetic nanohydroxyapatite. Contemp Clin Dent. 2012; 3: 433–436. 10.4103/0976-237X.107434 23633804 PMC3636833

[12] Chen H, Clarkson BH, Sun K, Mansfield JF. Self-assembly of synthetic hydroxyapatite nanorods into an enamel prism-like structure. J Colloid Interface Sci, 2005; 288: 97–103. 10.1016/j.jcis.2005.02.064 15927567

[13] Najibfard K, Ramalingam K, Chedjieu I, Amaechi BT. Remineralization of early caries by a nano-hydroxyapatite dentifrice. J Clin Dent. 2011; 22: 139–143. 22403978

[14] Vano M, Derchi G, Barone A, Covani U. Effectiveness of nano-hydroxyapatite toothpaste in reducing dentin hypersensitivity: a double-blind randomized controlled trial. Quintessence Int. 2014; 45: 703–711. 10.3290/j.qi.a32240 25019114

[15] Yuan P, Shen X, Liu J, Hou Y, Zhu M, Huang J et al Effects of dentifrice containing hydroxyapatite on dentinal tubule occlusion and aqueous hexavalent chromium cations sorption: A preliminary study. PLoS One. 2012; 7: 1–8. 10.1371/journal.pone.0045283 PMC3532500 23300511

[16] Bennat C, Müller-Goymann CC. Skin penetration and stabilization of formulations containing microfine titanium dioxide as physical UV filter. Int J Cosmet Sci. 2000; 22: 271–283. 10.1046/j.1467-2494.2000.00009.x 18503414

[17] Kiss B, Bíró T, Czifra G, Tóth BI, Kertész Z, Szikszai Z et al Investigation of micronized titanium dioxide penetration in human skin xenografts and its effect on cellular functions of human skin-derived cells. Exp Dermatol. 2008; 17: 659–667. 10.1111/j.1600-0625.2007.00683.x 18312389

[18] Shi H, Magaye R, Castranova V, Zhao J. Titanium dioxide nanoparticles: a review of current toxicological data, Part Fibre Toxicol. 2013; 10: 15 10.1186/1743-8977-10-15 23587290 PMC3637140

[19] Nabeshi H, Yoshikawa T, Matsuyama K, Nakazato Y, Matsuo K, Arimori A et al Systemic distribution, nuclear entry and cytotoxicity of amorphous nanosilica following topical application. Biomaterials 2011; 32: 2713–2724. 10.1016/j.biomaterials.2010.12.042 21262533

[20] Tay CY, Fang W, Setyawati MI, Chia SL, Tan KS, Hong CH et al Nano-hydroxyapatite and nano-titanium dioxide exhibit different subcellular distribution and apoptotic profile in human oral epithelium, ACS Appl Mater Interfaces. 2014; 6: 6248–6256. 10.1021/am501266a 24734929

[21] Frenkel ES, Ribbeck K. Salivary mucins in host defense and disease prevention. J Oral Microbiol. 2015; 7: 29759 10.3402/jom.v7.29759 26701274 PMC4689954

[22] Asikainen P, Ruotsalainen TJ, Mikkonen JJ, Koistinen A, Ten Bruggenkate C, Kullaa AM. The defence architecture of the superficial cells of the oral mucosa. Med Hypotheses. 2012; 78: 790–792. 10.1016/j.mehy.2012.03.009 22465465

[23] Lesniak A, Fenaroli F, Monopoli MP, Åberg C, Dawson KA, Salvati A. Effects of the presence or absence of a protein corona on silica nanoparticle uptake and impact on cells. ACS Nano. 2012; 7: 5845–5857. 10.1021/nn300223w 22721453

[24] Teubl BJ, Leitinger G, Schneider M, Lehr CM, Fröhlich E, Zimmer A et al The buccal mucosa as a route for TiO_2_ nanoparticle uptake. Nanotoxicology. 2015; 9: 253–261. 10.3109/17435390.2014.921343 24873758

[25] Teubl BJ, Meindl C, Eitzlmayr A, Zimmer A, Fröhlich E, Roblegg E. In-vitro permeability of neutral polystyrene particles via buccal mucosa. Small. 2013; 9: 457–466. 10.1002/smll.201201789 23112142

[26] Arun JL, Rani S, Manoj Kumar P. Buccal drug delivery system: History and recent developments. Asian J Pharm Clin Res. 2016; 19: 1–7.

[27] Squier CA, Kremer MJ. Biology of oral mucosa and esophagus, J Natl Cancer Inst Monogr. 2001; 29:7–15. 10.1093/oxfordjournals.jncimonographs.a003443 11694559

[28] Bragulla HH, Homberger DG. Structure and functions of keratin proteins in simple, stratified, keratinized and cornified epithelia, J. Anat. 2009; 214: 516–559. 10.1111/j.1469-7580.2009.01066.x 19422428 PMC2736122

[29] Elias PM. Stratum corneum defensive functions: an integrated view, J Invest Dermatol. 2005; 125: 183–200. 10.1111/j.0022-202X.2005.23668.x 16098026

[30] Bos JD, Meinardi MM. The 500 Dalton rule for the skin penetration of chemical compounds and drugs. Exp Dermatol. 2000; 9: 165–169. 10839713 10.1034/j.1600-0625.2000.009003165.x

[31] Kimura E, Todo H, Sugibayashi K. Skin penetration and safety of nanoparticles. Yakugaku Zasshi. 2012; 132: 319–324. (In Japanese) 22382836 10.1248/yakushi.132.319

[32] Collins LM, Dawes C. The surface area of the adult human mouth and thickness of the salivary film covering the teeth and oral mucosa. J Dent Res. 1987; 66: 1300–1302. 10.1177/00220345870660080201 3476596

[33] Dahl LK. A simple and sensitive histochemical method for calcium. Proc Soc Exp Biol Med. 1952; 80: 474–479. 14949089 10.3181/00379727-80-19661

[34] Kossa JV. Ueber die im Organismus künstlich erzeugbaren Verkalkungen. Beitr path Anat. 1901; 29: 163–202. (In German)

[35] Zaheer A, Lenkinski RE, Mahmood A, Jones AG, Cantley LC, Frangioni JV. In vivo near-infrared fluorescence imaging of osteoblastic activity. Nat Biotechnol. 2001; 19: 1148–1154. 10.1038/nbt1201-1148 11731784

[36] Kezic JM, Davey MP, Glant TT, Rosenbaum JT, Rosenzweig HL. Interferon-γ regulates discordant mechanisms of uveitis versus joint and axial disease in a murine model resembling spondylarthritis. Arthritis Rheum. 2012; 64: 762–771. 10.1002/art.33404 21987263 PMC3271189

[37] Michael S, Daniel G. International standardization in particle characterization for quality and safety assessment in particle technology. Procedia Eng. 2015; 102: 233–239.

[38] Suttiponparnit K, Jiang J, Sahu M, Suvachittanont S, Charinpanitkul T, Biswas P. Role of Surface Area, Primary Particle Size, and Crystal Phase on Titanium Dioxide Nanoparticle Dispersion Properties.Nanoscale Res Lett. 2010; 6: 27Available from:https://nanoscalereslett.springeropen.com/articles/10.1007/s11671-010-9772-1 27502650 10.1007/s11671-010-9772-1 PMC3211333

[39] Scheel J, Hermann M. Integrated risk assessment of a hydroxyapatite–protein-composite for use in oral care products: A weight-of-evidence case study. Regul Toxicol Pharmacol. 2011; 59: 310–323. 10.1016/j.yrtph.2010.11.003 21112362

[40] Larsen MJ, Pearce EI. A computer program for correlating dental plaque pH values, cH+, plaque titration, critical pH, resting pH and the solubility of enamel apatite. Arch Oral Biol. 1997; 42: 475–480. 9296266 10.1016/s0003-9969(97)00044-7

[41] Wang K, Zhou C, Hong Y, Zhang X. A review of protein adsorption on bioceramics. Interface Focus. 2012; 2: 259–277. 10.1098/rsfs.2012.0012 23741605 PMC3363020

[42] Humphrey SP, Williamson RT. A review of saliva: Normal composition, flow, and function. J Prosthet Dent. 2001; 85: 162–169. 10.1067/mpr.2001.113778 11208206

[43] Min L, Jian Z, Wei S, Yuan H. Developments of mucus penetrating nanoparticles. Asian Journal of Pharmaceutical Sciences. 2015; 10: 275–282.

[44] Lai SK, Wang YY, Hanes J. Mucus-penetrating nanoparticles for drug and gene delivery to mucosal tissues. Adv Drug Deliv Rev. 2009; 61: 158–171. 10.1016/j.addr.2008.11.002 19133304 PMC2667119

[45] Fröhlich E, Roblegg E. Models for oral uptake of nanoparticles in consumer products. Toxicology, 2012; 291: 10–17. 10.1016/j.tox.2011.11.004 22120540 PMC3273702

[46] Aljayyoussi G, Abdulkarim M, Griffiths P, Gumbleton M. Pharmaceutical Nanoparticles and the Mucin Biopolymer Barrier. Bioimpacts. 2012; 2: 173–174. 10.5681/bi.2012.029 23678457 PMC3648940

[47] Fröhlich E, Teubl BJ, Roblegg E. Titanium dioxide nanoparticles and the oral uptake-route. Bio Nano Mat. 2013; 14: 25–35.

[48] Arun JL, Rani S, Manoj KP. Buccal drug delivery system: History and recent developments. Asian J Pharm Clin Res. 2016; 19: 1–7.

[49] Creeth JE, Gallagher A, Sowinski J, Bowman J, Barrett K, Lowe S et al The effect of brushing time and dentifrice on dental plaque removal in vivo. J Dent Hyg. 2009; 83: 111–116. 19723429

[50] Hadrup N, Sharma AK, Loeschner K. Toxicity of silver ions, metallic silver, and silver nanoparticle materials after in vivo dermal and mucosal surface exposure: A review. Regul Toxicol Pharmacol. 2018; 98: 257–267. 10.1016/j.yrtph.2018.08.007 30125612

[51] Abdel-Gawad EI, Awwad S. Biocompatibility of intravenous nano-hydroxyapatite in male rats. Nat. Sci, 2010; 2: 60–68.

[52] Aoki H, Aoki H, Kutsuno T, Li W, Niwa M. An in vivo study on the reaction of hydroxyapatite-sol injected into blood. J Mater Sci Mater Med. 2000; 2: 67–72. 10.1023/a:1008993814033 15348049

